# Xanthurenic Acid Is the Main Pigment of *Trichonephila clavata* Gold Dragline Silk

**DOI:** 10.3390/biom11040563

**Published:** 2021-04-12

**Authors:** Masayuki Fujiwara, Nobuaki Kono, Akiyoshi Hirayama, Ali D. Malay, Hiroyuki Nakamura, Rintaro Ohtoshi, Keiji Numata, Masaru Tomita, Kazuharu Arakawa

**Affiliations:** 1 Institute for Advanced Biosciences, Keio University, Nihonkoku 403-1, Daihoji, Tsuruoka, Yamagata 997-0013, Japan; googoo.masa@gmail.com (M.F.); ciconia@sfc.keio.ac.jp (N.K.); hirayama@ttck.keio.ac.jp (A.H.); mt@sfc.keio.ac.jp (M.T.); 2 Systems Biology Program, Graduate School of Media and Governance, Keio University, Endo 5322, Fujisawa, Kanagawa 252-0882, Japan; 3 Biomacromolecules Research Team: RIKEN Center for Sustainable Resource Science, 2-1 Hirosawa, Wako, Saitama 351-0198, Japan; a.malay@riken.jp (A.D.M.); keiji.numata@riken.jp (K.N.); 4 Spiber Inc.: Mizukami 234-1, Kakuganji, Tsuruoka, Yamagata 997-0052, Japan; nakamura@spiber.jp (H.N.); rintaro_ohtoshi@spiber.jp (R.O.); 5 Faculty of Environment and Information Studies, Keio University, Endo 5322, Fujisawa, Kanagawa 252-0882, Japan

**Keywords:** spider, dragline, xanthurenic acid, UV tolerance, antibacteria

## Abstract

Spider silk is a natural fiber with remarkable strength, toughness, and elasticity that is attracting attention as a biomaterial of the future. Golden orb-weaving spiders (*Trichonephila clavata*) construct large, strong webs using golden threads. To characterize the pigment of golden *T. clavata* dragline silk, we used liquid chromatography and mass spectrometric analysis. We found that the major pigment in the golden dragline silk of *T. clavata* was xanthurenic acid. To investigate the possible function of the pigment, we tested the effect of xanthurenic acid on bacterial growth using gram-negative *Escherichia coli* and gram-positive *Bacillus subtilis*. We found that xanthurenic acid had a slight antibacterial effect. Furthermore, to investigate the UV tolerance of the *T. clavata* threads bleached of their golden color, we conducted tensile deformation tests and scanning electron microscope observations. However, in these experiments, no significant effect was observed. We therefore speculate that golden orb-weaving spiders use the pigment for other purposes, such as to attract their prey in the sunlight.

## 1. Introduction

Spider silk is utilized for a variety of purposes, including to catch prey, protect offspring, and act as a lifeline; it exhibits remarkable mechanical toughness, which is a combination of high tensile strength and elasticity [1]. Owing to its outstanding properties and biocompatible and biodegradable proteinaceous nature, spider silk has attracted attention as a potential tool in the commercial production of synthetic threads for medical and industrial applications [2,3,4,5], and various hosts are being explored for its synthetic production [6,7,8,9,10,11]. Protein composition [12,13] and the variability of the physical properties of spider silk have been extensively studied using the amino acid sequences encoded by spidroin genes as well as spider spinning processes [14,15,16]. However, the contribution of low molecular weight compounds to the properties of spider silk remains elusive.

Orb-weaving spiders harbor seven types of glands for silk production, namely, the major ampullate, minor ampullate, flagelliform, aggregate, cylindrical, aciniform, and piriform glands. Each gland, grouped into seven morphologically and biochemically distinct types, is specialized to make a particular type of silk [17]. Among these, the type of spider silk whose physical properties have been most investigated is the dragline silk produced in the major ampullate gland, which is used to build the framework and radii of spider webs and to prepare lifelines. However, studies of low molecular weight compounds have focused predominantly on the aqueous glue coating of the capture spiral produced in the aggregate gland [18]. Using nuclear magnetic resonance spectroscopy and chromatography, the compounds in glue droplets have been identified as glycoproteins, GABAmide, *N*-acetylputrescine, several amino acids, cholines, betaines, and salts, among others [19,20,21]. Variations in composition with regard to starvation and natural vs. laboratory conditions have also been studied in detail [22].

Thread color is a striking example of the effect of low molecular weight compounds in draglines. Golden orb-weaving spiders (*Trichonephila*), a genus of araneomorph spiders, produce bright yellow thread that absorbs light with wavelengths less than 500 nm [23]. Based on evidence that pigments in *Trichonephila clavipes* dragline silk are malleable and depend on light environments and that the yellow color functions as a lure [23,24,25], the color is mainly hypothesized to serve the purpose of attracting prey or camouflaging the orb web [26]. The yellow pigment of *T. clavipes* silk has been shown to be contained in the low molecular weight fraction in dissolved silks [27]. However, its actual composition has not been formally described until very recently. In fact, Holl and Henze in 1988 reported in a short conference abstract that *T. clavipes* silk contained a low amount of xanthurenic acid, a hydroxylated benzoquinone or napthoquinone and another quinone with similar properties as the constituents of yellow pigment [28]. Although this abstract had been cited for decades as the evidence of pigment, this is based on rudimentary technique of thin film chromatography (TLC), and the abstract lacks the details of the experiments and their results. However, recently Hsiung and colleagues reported the characterization of pigments in *T. clavipes* using Raman spectroscopy to be carotenoids [29]. The authors did not detect xanthurenic acid and other quinones, but the authors did not deny the existence of these compounds since carotenoids are highly Raman active and other signals can be overshadowed [29]. Quantitative high resolution metabolomics approach using liquid chromatography electrospray ionization quadrupole/time-of-flight mass spectrometry (LC-ESI-Q-TOF-MS) provides a comprehensive approach in detecting all low molecular weight compounds included in the sample, and therefore we here identified the pigment molecule of another *Trichonephila* spider, *T. clavata* to also provide evolutionary perspective of these pigments.

Pigments often serve dual purposes in addition to ecological functions; for example, lawsone from henna *Lawsonia inermis*, juglone from walnut and lapachol from alkanet have been reported to exhibit antibacterial and antifungal activity [30], as have some carotenoids [31,32,33]. Antimicrobial activity of spider silk was reported for common house spider *Tegenaria domestica* [34], but this work suggested protein element is responsible for this activity since proteinase K treatment reduced the antimicrobial activity. Common house spider weaves white silks, so the antimicrobial role of pigment in gold silks is an interesting area to explore. Furthermore, it is well known that pigment plays an important role in ultraviolet (UV) survival [35], and UV reflection is an important aspect of prey attraction by spider webs [36]. In terms of the ecological implication of the spider silk pigment, this prey attraction and web camouflage has been studied in detail [26], and the color has been reported to affect only the thermal properties of the silk [37]. However, prolonged exposure to UV radiation damages silk, and silkworm silk naturally use sericin coating to provide protection to UV damage by downregulating oxidative stress [38]. Pigments often serve not only as a shield by reflecting the light, but also as antioxidants [39]. To this end, we characterized the pigment of *T. clavata* dragline silk using high resolution mass spectrometry and examined whether this pigment contributes to other functions of the silk for antibacterial activity and protection from UV. This is, to the best of our knowledge, the first report of the characterization of the pigment molecule in *T. clavata* silk.

## 2. Materials and Methods

### 2.1. Pigment Extraction from Spider Silk

Dragline silk used in this study was collected (reel speed at 1.28 m/min) from *T. clavata* collected in Tsuruoka City, Japan in September 2015. Gold pigment was extracted from golden dragline silk of *T. clavata*; briefly, 1-mg gold threads were mixed and incubated with 200 µL of 1% sodium dodecyl sulfate (SDS) solution at room temperature for 30 min, and the extracted solution was collected in a new tube. 

### 2.2. LC- ESI-MS and LC-APCI-MS Conditions

Liquid chromatography mass spectrometry (LC- MS) analysis was performed using an Agilent 1200 series HPLC system coupled to an Agilent 6220 Accurate-Mass TOF LC/MS or quadrupole/time-of-flight (Q-TOF; Waters). The LC gradient maintained solvent B at 5% for 5 min, followed by a linear increase of B from 5% to 90% over 15 min. Solvent B was held at 90% for 5 min and the column was re-equilibrated at 5% B for 10 min. The flow rate was 200 µL/min and the column temperature was maintained at 40 °C. The injection volume was 1 µL. Data acquisition was performed with Agilent MassHunter Software (version B.05.00; Agilent Technologies, CA, USA). Electrospray ionization (ESI) was conducted using the following operation parameters: capillary voltage, 3500 V; nebulizer pressure 55 psig; drying gas flow rate, 12 L/min; drying gas temperature, 325 °C; fragmentor voltage, 150 V; skimmer voltage, 70 V; octopole 1 RF voltage, 250 V. Atomospheric Pressure Chemical Ionization (APCI) was conducted using operation parameters identical to ESI, except for drying gas temperature set to 325 °C. Mass spectra were recorded for m/z 50-1700 at a rate of 1 cycle/sec. Accurate mass spectra were obtained using a calibrant delivery system that enables continuous mass correction infusing two internal reference masses of purine (m/z 121.050873) and HP-0921 (m/z 922.009798) for ESI, m/z 68.995758 and m/z 966.000725 for APCI. The acquired raw data were processed using Agilent MassHunter Software (version B.06.00). LC-MS/MS analysis was performed using an Agilent 1290 series HPLC system coupled to an Agilent 6520 Accurate-Mass quadrupole-time-of-flight (Q-TOF) LC/MS. LC and ESI conditions were identical to those described in the previous section, except for the following changes: drying gas flow rate, 13 L/min; octopole 1 RF voltage, 750 V. The acquired raw data were processed with Agilent MassHunter Software (version B.04.00). 

### 2.3. LC-UV Conditions

Liquid chromatography ultraviolet detection (LC-UV) analysis was performed using an Agilent 1290 series HPLC system equipped with an Agilent G1315B DAD detector. The UV detector was set at 245 nm, and separation was performed on an Acquity UPLC BEH C18 column (2 × 100 mm, 1.7 µm; Waters, MA, USA). Mobile phases were (A) 0.1% formic acid and (B) methanol. LC conditions were identical to those described in the previous section. The extract of the pigment with 1% SDS was passed through an ultrafiltration membrane (nominal molecular weight limit of 3 kDa), and 10-fold diluted samples were used for LC analysis.

### 2.4. Quantitation of Xanthurenic Acid

First, 0.1% SDS was added to the dragline at 0.1 mL/mg in 1.5-mL microtubes. A stock solution of xanthurenic acid (Wako, Osaka, Japan) was prepared at a concentration of 10 mmol/L in 0.1 mol/L NaOH and stored at 4 °C until use. The working solution was prepared by diluting a stock solution with Milli-Q water at concentrations of 1, 5, 10, and 50 µmol/L. Xanthurenic acid quantitation was performed by applying the MS peak area of the sample to a calibration curve, which was measured at the beginning of the same sequence. 

For spectrophotometry of xanthurenic acid using the reduction of the Fe3+—TPTZ (2,4,6-Tris (2-pyridyl)-1,3,5-triazine) complex, 0.1% SDS was added to the dragline at 0.04 mL/mg in 1.5-mL microtubes. The mixtures were vortexed for 3 min, followed by incubation at 4 °C for 16 h. The extracts were centrifuged at 12,000 rpm for 5 min. The supernatants were removed and diluted as appropriate for spectrophotometric analyses. Then, 0.02 mL of extracts were added to a solution containing 0.5 mL of acetate buffer, pH 4.0, 0.02 mL of 0.1 N NaOH, 0.06 mL of TPTZ (Sigma-Aldrich Japan; Tokyo, Japan), and 0.02 mL of FeCl_3_ solution. The extracts were mixed well and incubated at room temperature for 15 min. The absorbance of each tube was immediately measured at 593 nm.

### 2.5. Antibacterial Activity Test

Wild-type *Escherichia coli* (W3110) and *Bacillus subtilis* (str. 168) were cultivated in M9 minimal medium (7 mg/mL K_2_HPO_4_, 3 mg/mL KH_2_PO_4_, 0.5 mg/mL NaCl, 1 mg/mL NH_4_Cl, 1 mg/mL glucose, 1 mM MgSO_4_•7H_2_O, 14.7 µg/mL CaCl_2_) containing 10 µg/mL thiamine, and Spizizen minimal medium [40] containing 50 µg/mL tryptophan, respectively. Each medium was prepared with Tris-HCl (pH 8) instead of H_2_O because pH affects the solubility of xanthurenic acid (Wako, Osaka, Japan). Overnight cultures (0.8%) were diluted in 20 mL fresh medium containing 0 µM, 10 µM, 100 µM or 1 mM xanthurenic acid. Growth was monitored by measuring OD600 every hour using a Pearl NanoPhotometer (Implen, Munich, Germany).

### 2.6. UV Tolerance Assay

Dragline silk was extracted from a single female *T. clavata* spider at a reeling rate of 126 mm s^−1^. Silk segments approximately 10 cm in length were secured at either end with adhesive tape and immersed in one of three conditions for 1 h: deionized water (control), 1% SDS or 20 mM sodium phosphate buffer, pH 11. Samples were subsequently washed with deionized water and allowed to air-dry for three days. Each fiber was cut into several segments, each of which was attached to a 20-mm square cardboard frame with an aperture of 5 mm using 95% cyanoacrylate glue. The samples were then divided into a control group (no treatment) and a test group that was subjected to UV irradiation at 302 nm for 13 h using a benchtop 3UV trans-illuminator (UVP, CA, USA). Each sample was subjected to tensile deformation using an EZ-LX universal tester (Shimadzu, Kyoto, Japan) with a 1 N load cell at a constant strain rate of 0.033 s^−1^. The force-displacement data were combined with fiber diameter measurements for each sample to calculate the values for ultimate tensile strength (stress), elongation to break (strain) and Young’s modulus. Toughness values were calculated by computing the area under the stress-strain curves using customized software. Measurement of silk fiber diameter and visualization of surface morphology was performed using scanning electron microscopy (SEM) at 5 kV using a JCM 6000 (JEOL Ltd., Tokyo, Japan) with samples sputter-coated with gold using a JEOL Smart Coater (JEOL Ltd.) prior to visualization.

## 3. Results

### 3.1. Characterization of the Gold Pigment

**Figure 1:**
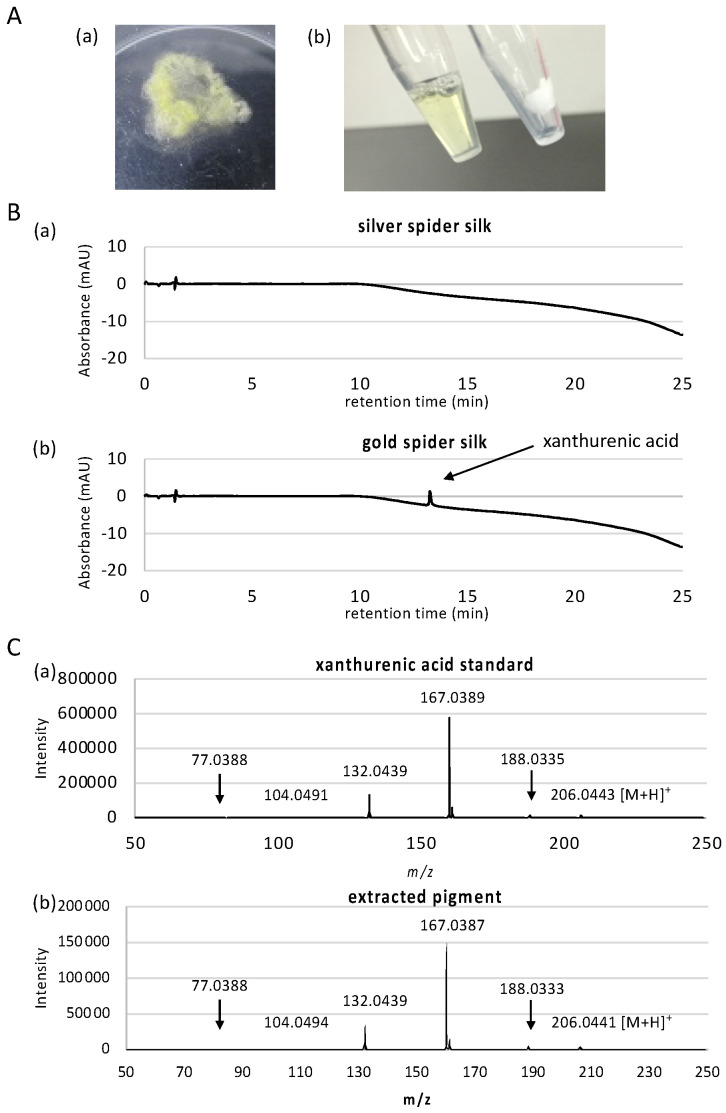
Extraction and measurement of pigment from dragline silk. (**A**). Dragline silk was collected from *Trichonephila clavata* (**a**) and (**b**) destained with 1% sodium dodecyl sulfate (SDS) solution (right tube; destained silk, left tube; extracted solution containing gold pigment). (**B**). LC chromatograms of silver spider silk (**a**) and (**b**) gold spider silk. A peak at 13.27 min corresponding to xanthurenic acid was only observed in the gold dragline. (**C**). MS/MS spectrum of xanthurenic acid standard (**a**) and extracted pigment (**b**) peak specifically detected in gold sample. Both samples show identical spectra.

To identify the gold pigment, we first considered the extraction conditions for the pigment from the thread of *T. clavata*. We did not use silk gland for this purpose because the cells in silk glands produce a multitude of unrelated metabolites and therefore it is more difficult to pinpoint the pigment molecule. We found that 1% SDS and Tris (pH 11) buffer successfully removed all visible gold pigment from the golden thread (Figure 1A).

When we forcibly silked multiple individuals of *T. clavata* from the wild, a small number of individuals occasionally produced pigment-less silver silks. We therefore used these silver silks as a pigment-less control to identify the pigment molecule responsible for the gold color. To investigate the pigment constituent differences between gold and silver spider silk, we then performed high resolution LC-MS analysis using two ionization techniques ESI and APCI for comprehensive identification of the pigment for all molecules contained in the silk between 50 to 1700 m/z. LC-ESI-MS identified a total of 6401 and 6414 peaks in silver and gold silks, respectively, combining positive and negative scan modes. LC-APCI-MS identified a total of 398 and 364 peaks in silver and gold silks, respectively, combining positive and negative scan modes. After filtering for peaks with significant signal-to-noise ratio, and for peaks only overrepresented in either of the samples, only one peak remained in LC-APCI-MS result that is overrepresented in the silver silk, and 38 peaks remained in LC-ESI-MS result, of which 15 peaks were overrepresented in the gold silk (Table 1 and see Supplementary Figure S1 for comprehensive peak data). Of the 15 peaks overrepresented in the gold silk, 7 of them were related to xanthurenic acid. Xanthurenic acid had the maximum peak area, more than triple of the second largest peak, implying that this is the major pigment of the silk. Since we employed high resolution mass spectroscopy using ESI-Q-TOF, elemental composition of the peaks can be accurately determined based on exact mass and isotope distribution of elements. However, to further confirm that the compound is actually xanthurenic acid, we performed MS/MS fragment analysis. MS/MS spectra clearly showed that the peak is actually xanthurenic acid (Figure 1C). 

Although the use of two ionization techniques of ESI and APCI should be able to ionize and detect pigment molecules comprehensively, we further performed UV-based detection where UV detector was used in place of the ESI-Q-TOF instrument, in case ionization was difficult. A previous study reported the presence of p-benzoquinone and naphthoquinone, as well as carotenoids, which are difficult to be ionized. As seen in Figure 1C and Figure S2, LC-UV detection also clearly detected xanthurenic acid in high abundance only in gold silk sample (peak at 13.27 min), confirming that this is the primary pigment molecule in *T. clavata*. Moreover, no other compounds were detected in LC-UV analysis. The second most abundant peak in LC-ESI-MS analysis was determined to have elemental composition of C_10_H_6_NO_6_ (Table 1), where direct search of the ChemSpider database (http://www.chemspider.com/, accessed on 24 November 2016) using this formula suggested diazodiphenoquinone, or nicotine blue, as a possible pigment candidate. However, MS/MS analysis of this peak showed the existence of hydroxyl as well as carboxyl groups in this compound (Figure S3), that are lacking in nicotine blue. Xanthurenic acid contains one carboxyl end and two hydroxyl groups, and its direct precursor molecule 3-hydroxy-L-kynurenine also contain carboxyl and hydroxyl groups, and two nitrogen atoms. Therefore, peaks other than those confirmed to be related to xanthurenic acid are likely to be related compounds to xanthurenic acid. Xanthurenic acid has been identified as a pigment in insects [41], synthesized using the ommochrome biosynthesis pathway starting from the oxidation of tryptophan to kynurenine and 3-hydroxykynurenine [42]. Ommochrome pigments are the main hypodermal pigments in spiders, and the intermediate metabolites kynurenine and 3-hydroxykynurenine are both yellow pigments [43]. Since both compounds were absent in our mass spectrometry results, xanthurenic acid is the primary pigment in *T. clavata* draglines, adding a new compound to the Class III ommochromes used by spiders [44]. Xanthurenic acid, therefore, is presumably synthesized by the transamination of 3-hydroxykynurenine using a branch pathway of ommochrome biosynthesis as in orthologous insect pathways [45]. Utilization of the ommochrome pathway for pigment production supports previous hypotheses indicating a function in avoiding excess accumulation of highly toxic tryptophan [46], which may partly explain the malleability of dragline color in *Trichonephila* [27].

Interestingly, carotenoids were not detected in our analysis, contrary to previous report of the close relative *T. clavipes*. Although our LC-MS scanned molecules up to 1700 m/z which should include carotenoid compounds (for example, yellow carotenoids zeaxanthin and beta-cryptoxanthin are around 550 m/z), the difference in the spectroscopy methodologies (LC-MS and LC-UV in this work, Raman spectroscopy in the previous work) could be the one possible reason for the different results. However, use of diverse pigments have been reported for spider silks, therefore the differences could also be due to the specific adaptations of *T. clavata* and *T. clavipes*.

Since we identified xanthurenic acid to be the primary pigment of golden *T. clavata* silk, we next quantified the abundance of this pigment contained in the silk. A standard curve ranging from 1 to 50 µmol/L using xanthurenic acid standard was constructed to calculate the sample concentration. The samples were prepared in triplicate, and each sample was analyzed by LC-Q-TOF MS. The average concentration of xanthurenic acid in gold spider silk was 181 µmol/L in 100 µL 0.1% SDS per 1 mg thread, which corresponds to 0.371% of the weight of the dragline. As an alternative confirmation of the amount of xanthurenic acid, we used spectrophotometry coupled to the reduction of the Fe^3+^—TPTZ complex [47] by xanthurenic acid (Supplemental Figure S4). This measurement resulted in a 260 µM concentration in a 40 µL 0.1% SDS per 1 mg thread, corresponding to 0.213% weight. Quantification is usually more accurate in mass spectrometry than spectrophotometry; however, the difference may also be due to the inherent variability among samples. We therefore consider the amount of xanthurenic acid in *T. clavata* dragline to be 0.2–0.4%.

### 3.2. Growth Inhibition Effect of Xanthurenic Acid

**Figure 2:**
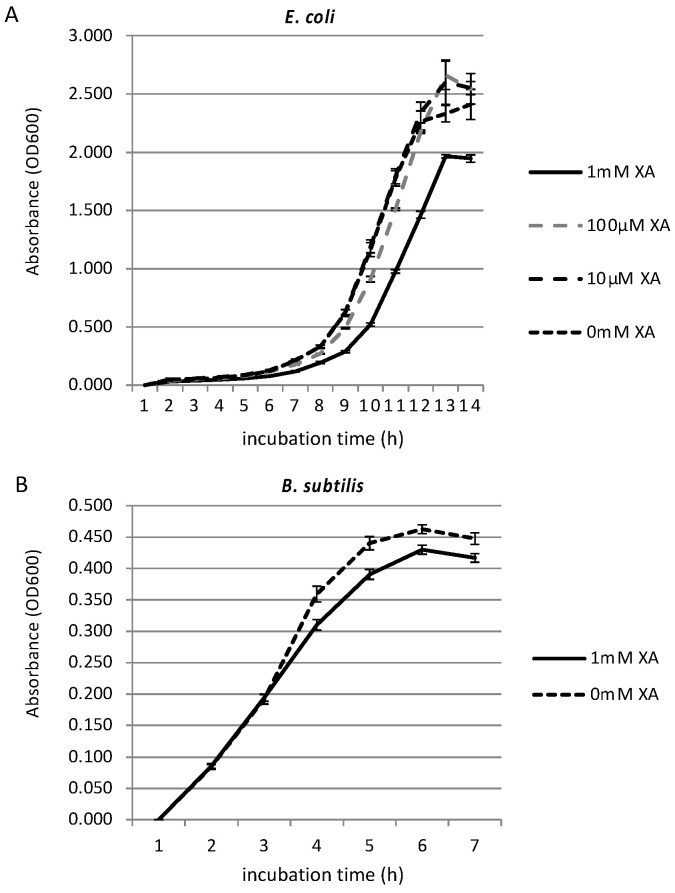
Growth inhibition effect of xanthurenic acid. Growth curves of *E. coli* (**A**) and *B. subtilis* (**B**) in LB medium with different concentrations of xanthurenic acid (XA). In both species, 1 mM xanthurenic acid has significant inhibitory effects (Welch *t*-test *p* < 0.05).

A previous study showed that spider silk has an effect on bacterial growth [34]. Since some natural pigments have antimicrobial activity [31,32], and since xanthurenic acid has been shown to have inhibitory effect on human glioma cells [48], we investigated whether xanthurenic acid also has an effect on common bacterial growth. We tested two types of bacterial cultures, gram-negative *E. coli* and gram-positive *B. subtilis*, in order to investigate the effects on broader spectrum of bacteria. We investigated the growth inhibition effect of xanthurenic acid against *E. coli* and *B. subtilis* grown in the appropriate medium with shaking at 37 °C by measuring OD600 every hour. Figure 2 shows the growth curves of *E. coli* (A) and *B. subtilis* (B) in minimal medium with different concentrations of xanthurenic acid. We first investigated the growth-inhibiting effect of xanthurenic acid against *E. coli* at concentrations of 10 µM, 100 µM, and 1 mM. The tests at the selected concentrations and negative controls were replicated three times. As shown in Figure 2A, for 10 and 100 µM xanthurenic acid, there was little or no inhibition effect, with the growth curve almost overlapping the negative control (0 mM xanthurenic acid). However, the growth of *E. coli* in medium containing 1 mM xanthurenic acid was suppressed compared to the negative control and 10 and 100 µM xanthurenic acid. Subsequently, a similar experiment was conducted using *B. subtilis* (Figure 2B). As a result, the growth of *B. subtilis* incubated with 1 mM xanthurenic acid was slightly suppressed compared to the negative control. These results revealed that xanthurenic acid has a certain amount of antibacterial activity against gram-positive and -negative bacteria, effective for a broad spectrum of bacteria. Because *T. clavata* replaces webs rapidly [49], strong antibacterial activity may not be necessary.

### 3.3. UV Irradiation to Dragline Silk

**Figure 3:**
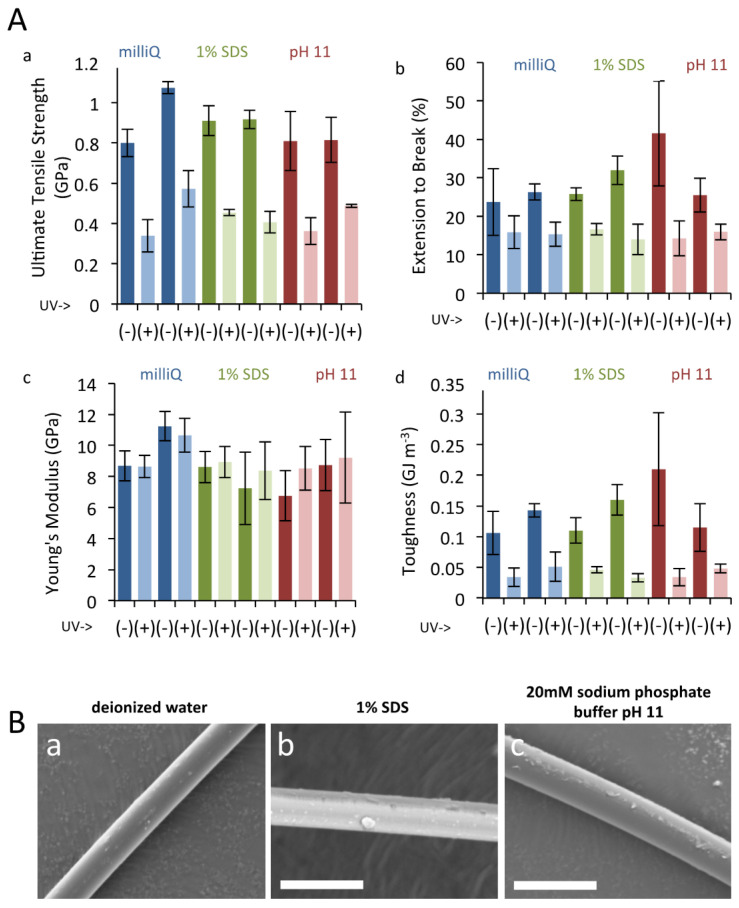
UV irradiation to dragline silk. (**A**). Tensile deformation test results of *T. clavata* dragline silk. Calculated values for ultimate tensile strength (**a**), elongation to break (**b**), Young’s modulus (**c**), and toughness (**d**) are shown. Results for two individual replicates are presented for each of the three pigment extraction treatments (control, 1 % SDS, and pH 11), with and without subsequent UV irradiation. Error bars represent standard deviation values for three separate experiments. (**B**). SEM results for *T. clavata* dragline silk treated with (**a**) deionized water, (**b**) 1 % SDS, and (**c**) 20 mM sodium phosphate buffer pH 11. Scale bars represent 10 µm.

Prolonged exposure to UV radiation damages silk, and silkworm silk naturally use sericin coating to provide protection to UV damage by downregulating oxidative stress [38]. We therefore tested the protective effect of xanthurenic acid against UV damage, by exposing natural gold silk and bleached silk to UV. Silk was bleached in two ways; namely, by immersion in 1% SDS or in strong alkaline (pH 11) conditions. The results of tensile deformation tests are summarized in Figure 3A. Prolonged UV treatment led to a drastic reduction in final tensile strength and elongation to break of the individual fibers and, consequently, to significantly reduced toughness values. The effect on the initial elastic modulus (Young’s modulus) was minimal. These findings are consistent with previous reports [50]. Comparison of results obtained from control silk fibers and those subjected to pigment extraction via immersion in 1% SDS or strong alkaline (pH 11) conditions, however, revealed that UV treatment led to a comparable reduction in mechanical properties among the different treatments. Thus, no correlation could be drawn between the presence of the yellow pigment in the dragline silk and the degree of physical protection afforded against UV irradiation damage. SEM measurements likewise revealed no apparent changes in the surface morphology of the silk fibers upon treatment with 1% SDS or pH 11 solutions (Figure 3B). From the results of these tensile tests, the yellow pigment does not appear to contribute to UV tolerance or mechanical performance. A lack of effect of these pigments on mechanical properties is in line with previous reports using silk produced in captivity [27]. However, since our experimental procedures presumably washed away the majority of small molecules in addition to the pigments, due to the use of strong detergent, we speculate that low molecular weight compounds have a negligible effect on the mechanical properties of dragline silk and can probably be ignored when designing synthetic spider silk.

## 4. Conclusions

By coupling liquid chromatography with high resolution ESI-MS, APCI-MS, and UV detector, and further confirming the structure by MS/MS spectrometric analysis, the major pigment of golden dragline derived from *T. clavata* was identified as xanthurenic acid. Xanthurenic acid is presumably produced through the branch pathway of the ommochrome biosynthesis pathway. We then investigated the effect of bacterial growth and UV tolerance on the bleached threads, but in both experiments, significant, but only marginal effect was observed. We therefore speculate that the primary function of the pigment is for other ecological purposes, such as prey attraction as previously reported.

## Figures and Tables

**Table 1:** Differentially detected peaks in LC-ESI-MS analysis of gold and silver silks.

	ID	Migration Time	Average m/z	Average Area		Formula	Comment
ESI_POS	25	13.25	81.98547	10,800.3	Silver only		ID416-related
	32	13.23	86.99120	10,614.0	Silver only		ID416-related
	39	13.26	89.05266	24,523.0	Silver only		ID416-related
	61	13.25	96.98379	19,043.3	Silver only		ID416-related
	64	12.37	99.04526	31,315.7	Silver only		
	67	4.71	100.07710	22,357.3	Silver only		
	74	8.62	103.07697	23,533.3	Silver only		
	82	13.24	105.03710	11,356.7	Silver only		
	121	2.88	117.09238	12,281.3	Silver only		
	145	13.24	129.98900	51,675.7	Silver only		ID416-related
	229	9.49	160.04129	36,029.7	Gold only		xanthurenic acid-HCOOH
	252	8.62	165.11397	20,5405.3	Silver only	C7H16O4	
	258	8.62	166.11690	17,431.0	Silver only		ID252 isotope
	323	8.63	182.14073	74,372.3	Silver only		ID252 plus NH4+
	346	8.62	187.09587	11,368.7	Silver only		
	353	9.49	188.03569	11,801.0	Gold only		xanthurenic acid-H2O
	380	10.81	193.14507	20,329.3	Silver only		
	416	13.24	201.12398	26,460.7	Silver only		
	457	9.49	206.04450	677,591.0	Gold only	C10H7NO4	xanthurenic acid
	470	9.49	207.05003	72,818.0	Gold only		xanthurenic acid 13C-isotope
	479	9.49	208.05075	9736.3	Gold only		xanthurenic acid 13C*2-isotope
	501	8.62	210.17206	14,782.7	Silver only		
	532	10.83	215.12811	13,143.3	Silver only		
	547	12.37	219.12465	32,967.3	Silver only		ID643-NH3
	604	9.49	228.02776	9588.7	Gold only		
	643	12.37	236.15169	88,752.7	Silver only	C10H21NO5	
	652	12.37	237.15506	11,120.0	Silver only		ID643 isotope
	679	12.37	241.10661	10,978.3	Silver only		
	697	13.58	245.15811	9542.3	Silver only		
	737	11.22	251.03191	117,229.7	Gold only	C10H6N2O6	peak tailing
	1076	4.98	301.05057	11,205.0	Gold only		
	3407	17.29	904.76282	10,659.7	Gold only		
ESI_NEG	304	9.52	160.04257	105,782.3	Gold only		xanthurenic acid-CO2
	332	9.53	161.04552	10,576.7	Gold only		ID304 isotope
	894	11.23	205.02788	34,258.3	Gold only		ID1209-related
	895	9.52	205.03566	36,281.3	Gold only		xanthurenic acid isotope
	1209	11.22	249.01787	177,504.3	Gold only	C10H6N2O6	same as pos ID737
	1789	9.52	272.01950	9950.7	Gold only		

## Data Availability

All data associated with the manuscript is available upon request from the authors.

## Supplementary Materials

The following are available online at https://www.mdpi.com/article/10.3390/biom11040563/s1, Figure S1: Detailed spectra for each of differentially detected peaks, Figure S2: LC-UV detection of pigments, Figure S3: MS/MS spectra of C10H6N2O6 compound, Figure S4: Quantitation of xanthurenic acid by reduction of Fe3+ - TPTZ complex.
