# Supplementary material for: Xanthurenic Acid Is the Main Pigment of Trichonephila clavata Gold Dragline Silk
Source: Biomolecules. 2021 Apr 12;11(4):563. doi: 10.3390/biom11040563 (PMC8070366; doi:10.3390/biom11040563)
Supplement: Supplementary file 1 [file biomolecules-11-00563-s001.pdf]

APCI_POS / ID296, Ave m/z 183.1021, Cor MT 13.33

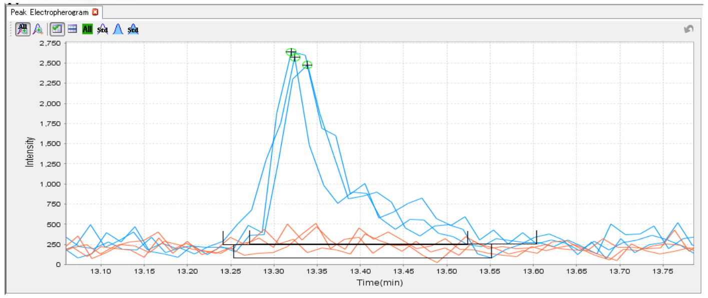

ESI_POS / ID61, Ave m/z 96.98379, Cor MT 13.25

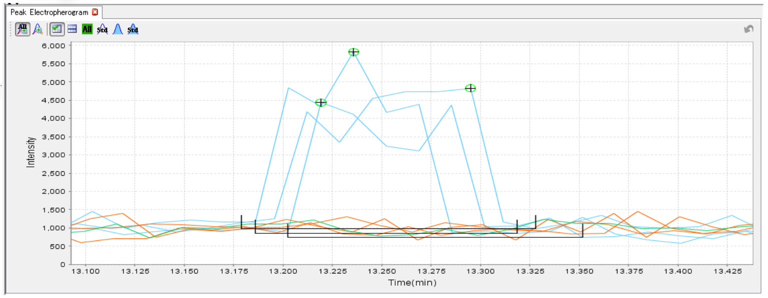

ESI_POS / ID25, Ave m/z 81.98547, Cor MT 13.25

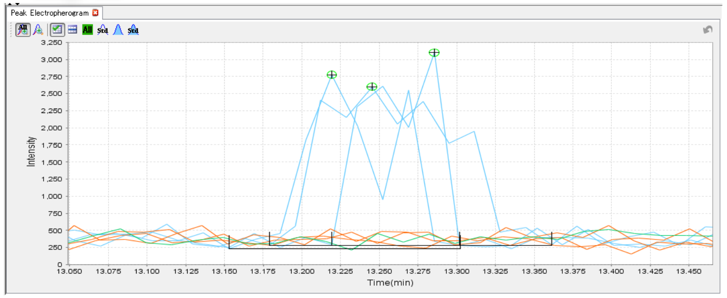

ESI_POS / ID64, Ave m/z 99.04526, Cor MT 12.37

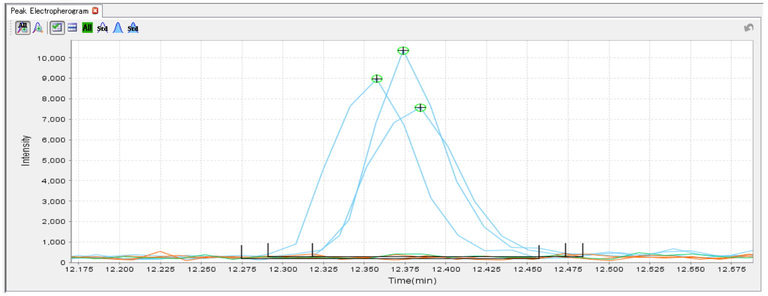

ESI_POS / ID32, Ave m/z 86.99120, Cor MT 13.23

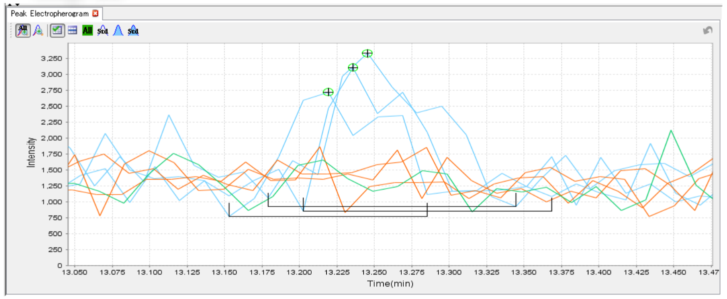

ESI_POS / ID68, Ave m/z 100.0771, Cor MT 4.71

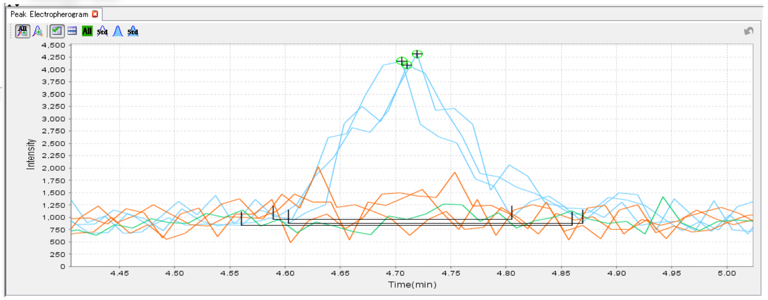

ESI_POS / ID39, Ave m/z 89.05266, Cor MT 13.26

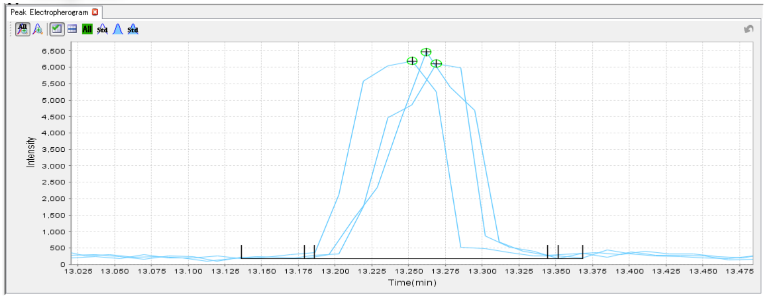

ESI_POS / ID74, Ave m/z 103.07697, Cor MT 8.62

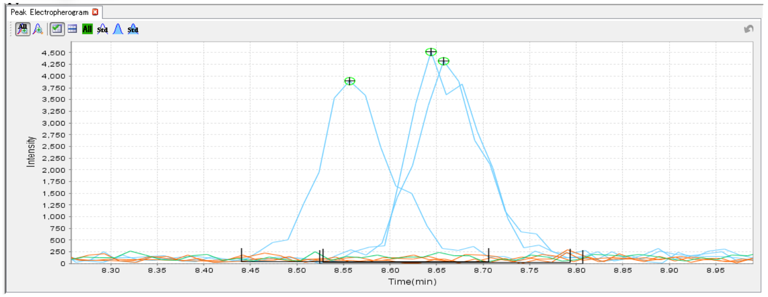

Figure S1. Detailed spectra for each of differentially detected peaks

Figure S1

ESI_POS / ID82, Ave m/z 105.03710, Cor MT 13.24

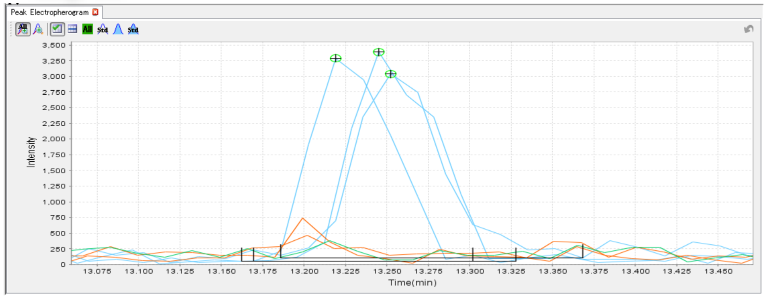

ESI_POS / ID121, Ave m/z 117.09238, Cor MT 2.88

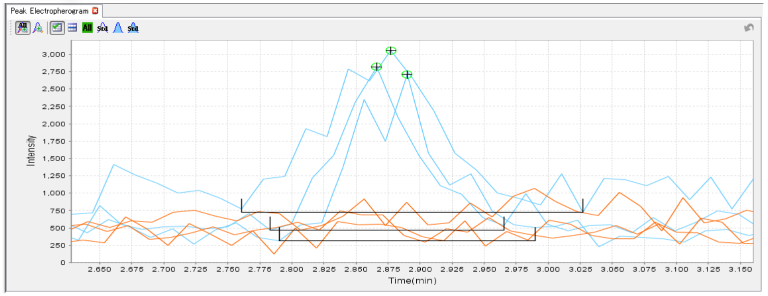

ESI_POS / ID145, Ave m/z 129.98900, Cor MT 13.24

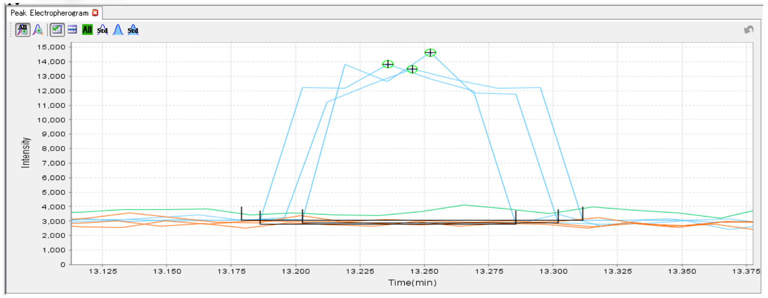

ESI_POS / ID229, Ave m/z 160.04129, Cor MT 9.49

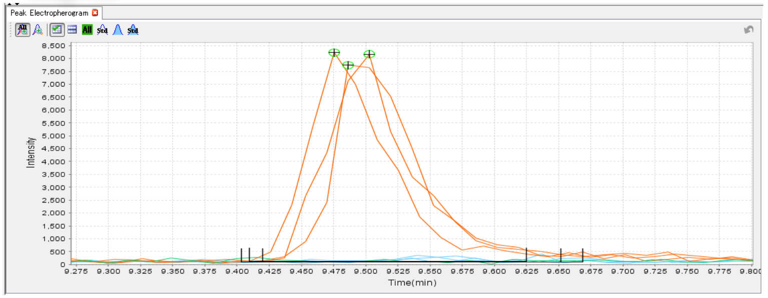

ESI_POS / I252, Ave m/z 165.11397, Cor MT 8.62

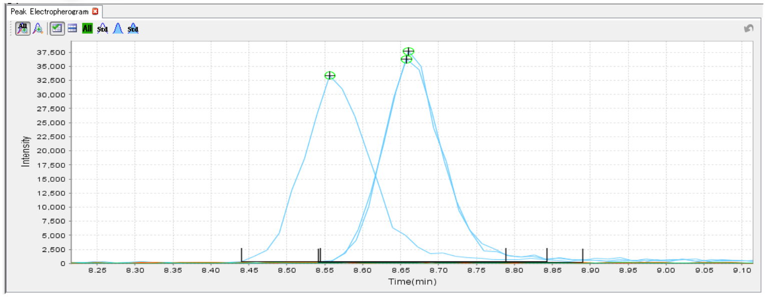

ESI_POS / ID258, Ave m/z 166.11690, Cor MT 8.62

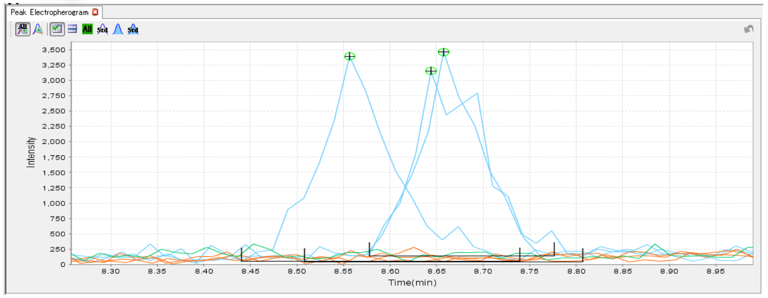

ESI_POS / ID323, Ave m/z 182.14073, Cor MT 8.63

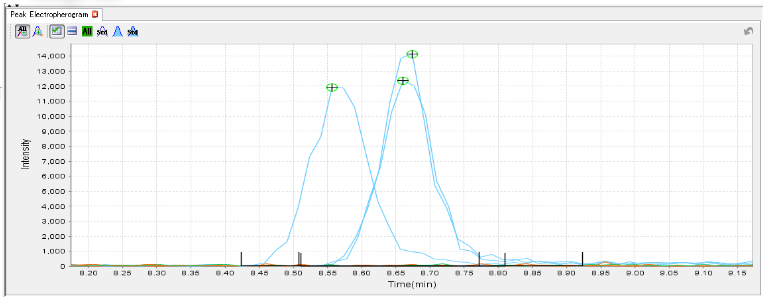

ESI_POS / ID346, Ave m/z 187.09587, Cor MT 8.62

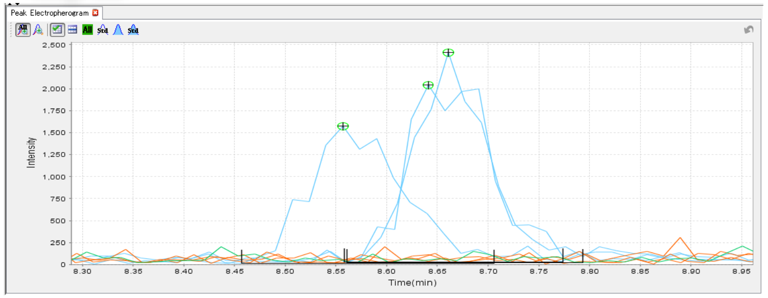

Figure S1. Detailed spectra for each of differentially detected peaks

Figure S1
continued 2/5

ESI_POS / ID353, Ave m/z 188.03569, Cor MT 9.49

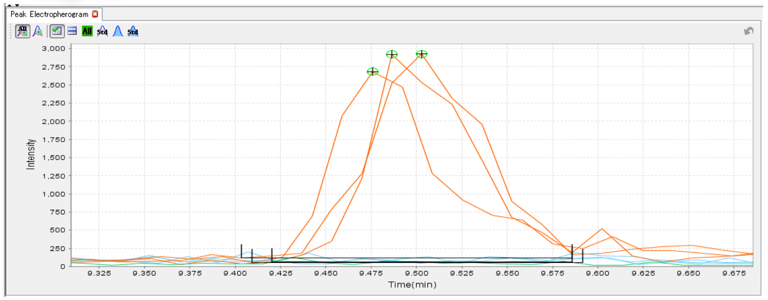

ESI_POS / ID380, Ave m/z 193.14507, Cor MT 10.81

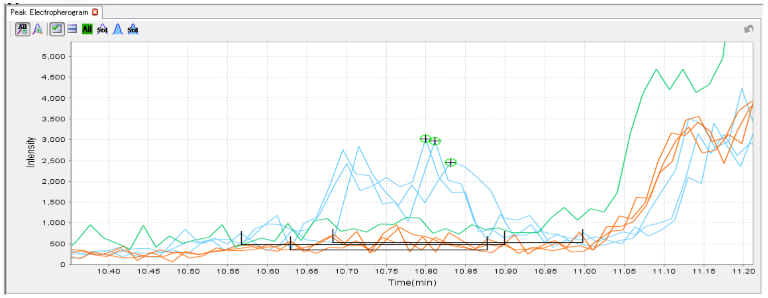

ESI_POS / ID416, Ave m/z 201.12398, Cor MT 13.24

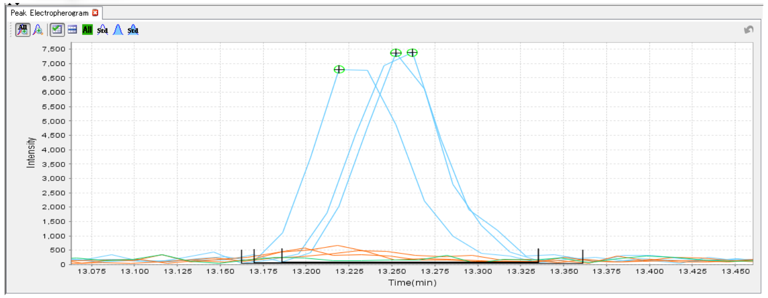

Xanthurenic acid

ESI_POS / ID470, Ave m/z 207.05003, Cor MT 9.49

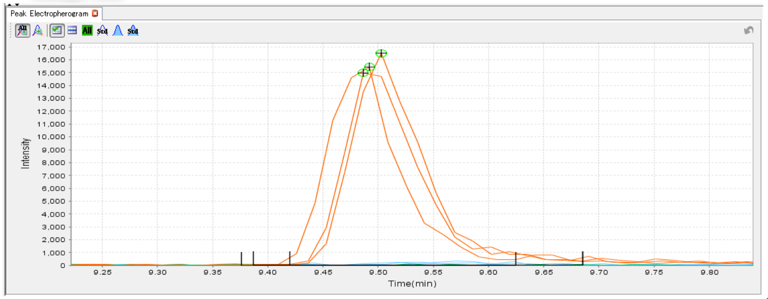

ESI_POS / ID479, Ave m/z 208.05075, Cor MT 9.49

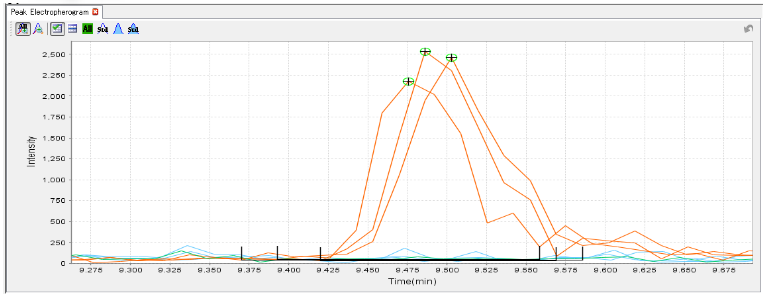

ESI_POS / ID501, Ave m/z 210.17206, Cor MT 8.62

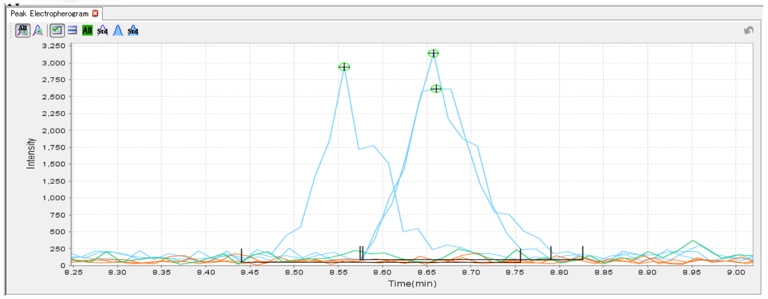

ESI_POS / ID532, Ave m/z 215.12811, Cor MT 10.83

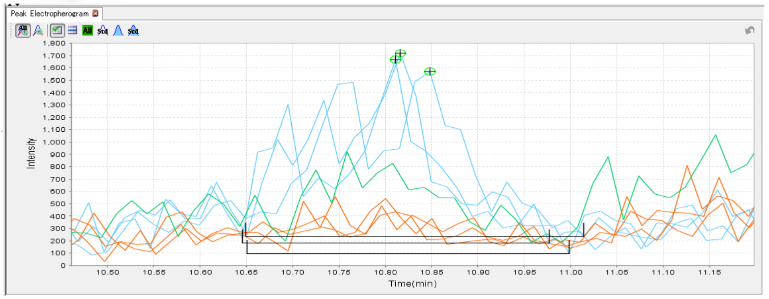

ESI_POS / ID547, Ave m/z 219.12465, Cor MT 12.37

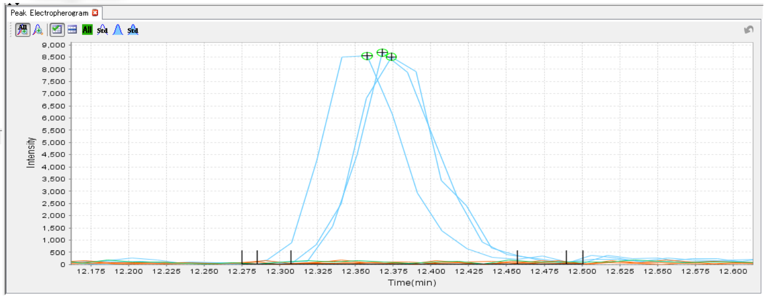

Figure S1. Detailed spectra for each of differentially detected peaks

Figure S1 continued 3/5

ESI_POS / ID604, Ave m/z 228.02776, Cor MT 9.49

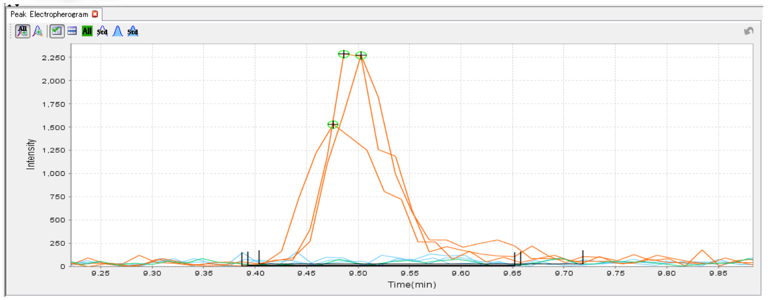

ESI_POS / ID643, Ave m/z 236.15169, Cor MT 12.37

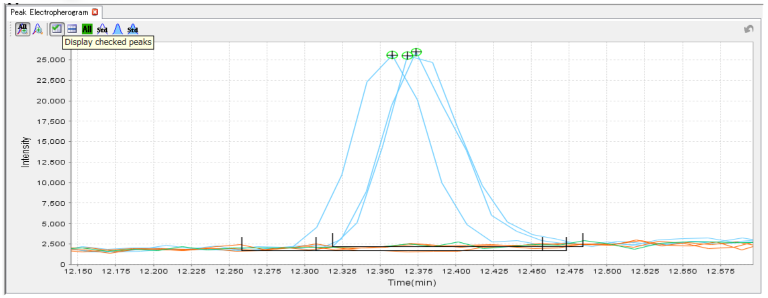

ESI_POS / ID652, Ave m/z 237.15506, Cor MT 12.37

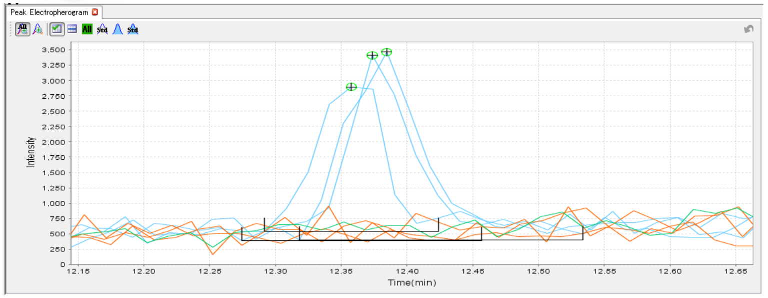

ESI_POS / ID679, Ave m/z 241.10661, Cor MT 12.37

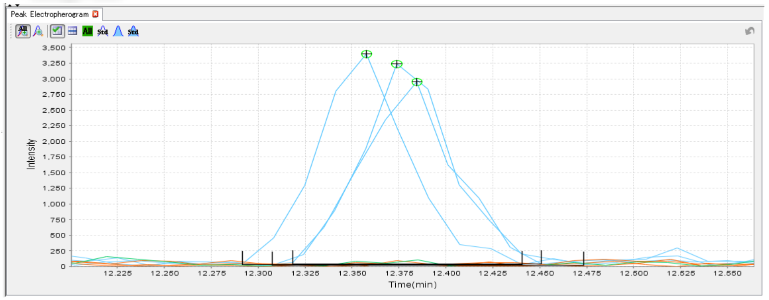

ESI_POS / ID697, Ave m/z 245.15811, Cor MT 13.58

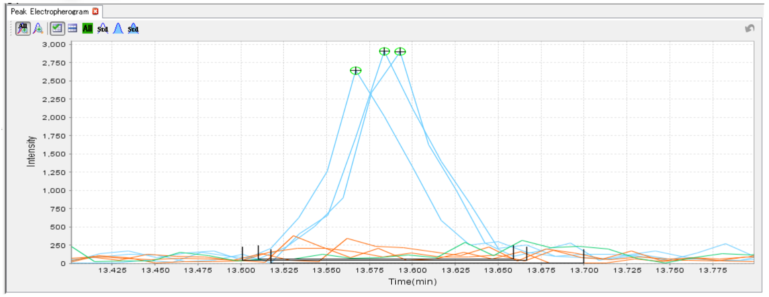

ESI_POS / ID737, Ave m/z 251.03191, Cor MT 11.22

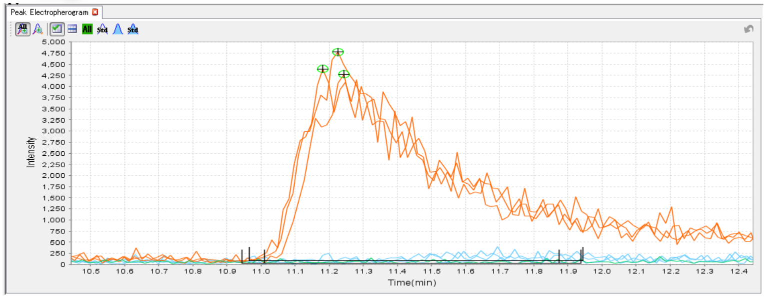

ESI_POS / ID1076, Ave m/z 301.05057, Cor MT 4.98

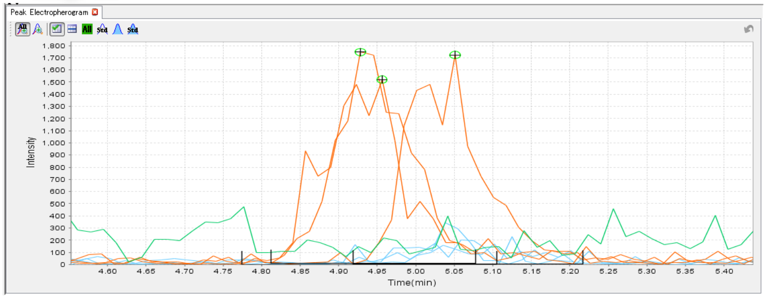

ESI_POS / ID3047, Ave m/z 904.76282, Cor MT 17.29

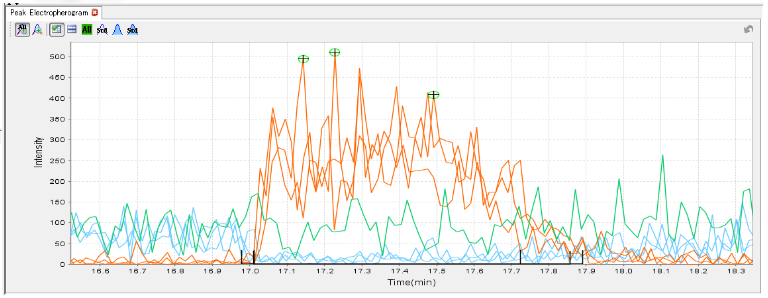

Figure S1. Detailed spectra for each of differentially detected peaks

Figure S1
continued 4/5

ESI_NEG / ID304, Ave m/z 160.04257, Cor MT 9.52

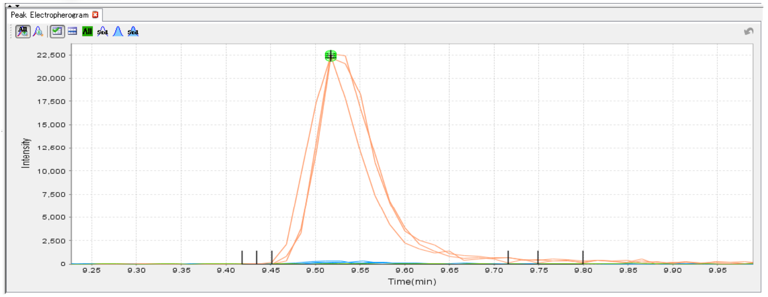

ESI_NEG / ID1209, Ave m/z 249.01787, Cor MT 11.22

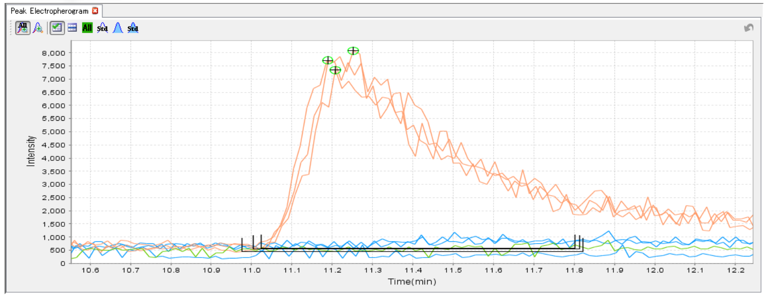

ESI_NEG / ID332, Ave m/z 161.04552, Cor MT 9.53

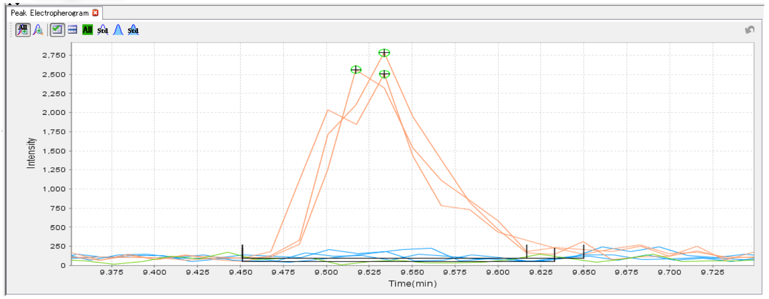

ESI_NEG / ID737, Ave m/z 272.01950, Cor MT 9.52

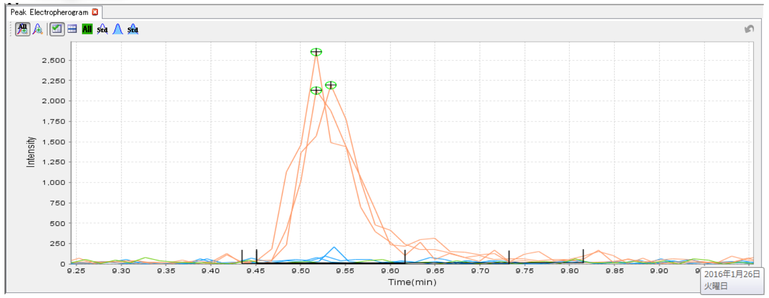

ESI_NEG / ID894, Ave m/z 205.02788, Cor MT 11.23

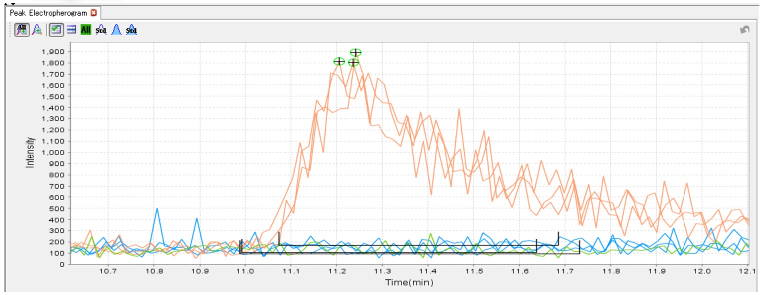

Xanthurenic acid

ESI_NEG / ID895, Ave m/z 205.03566, Cor MT 9.52

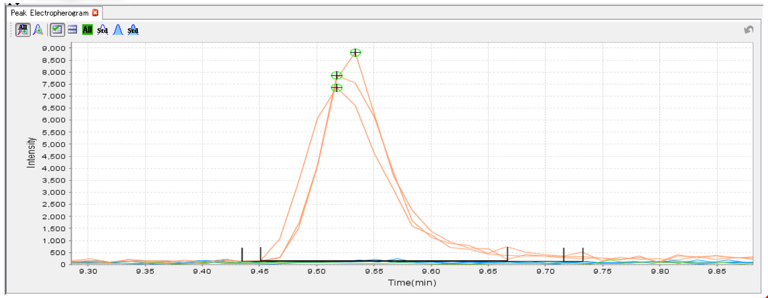

Figure S1. Detailed spectra for each of differentially detected peaks

Figure S1 continued 5/5

100 μM STD mix

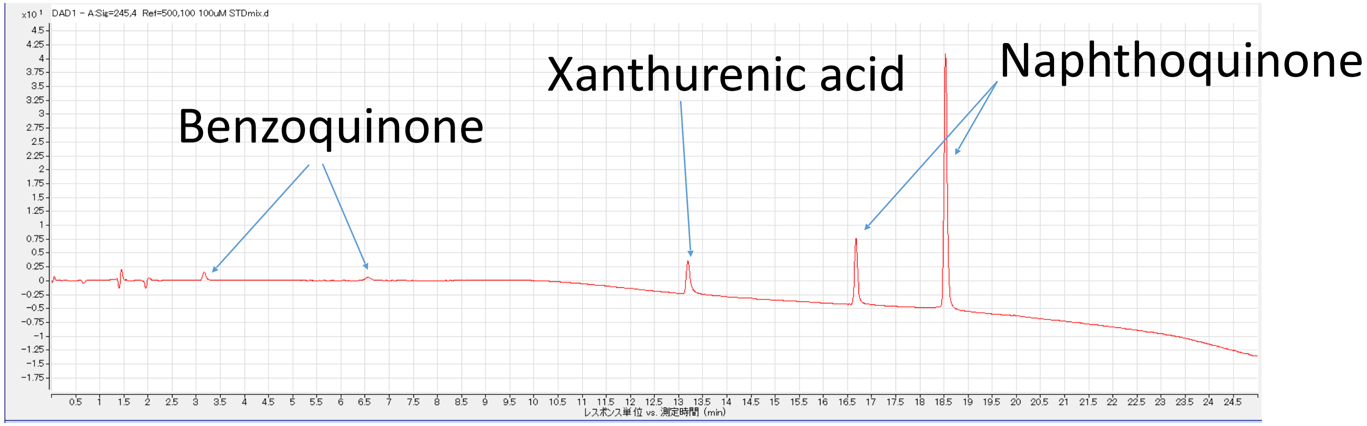

10-fold diluted silver spider silk extract

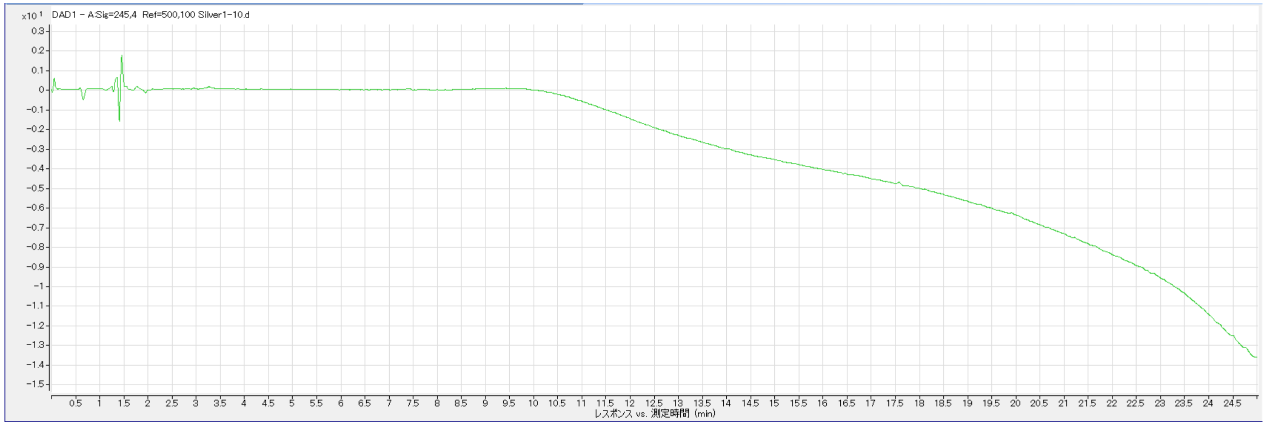

10-fold diluted gold spider silk extract

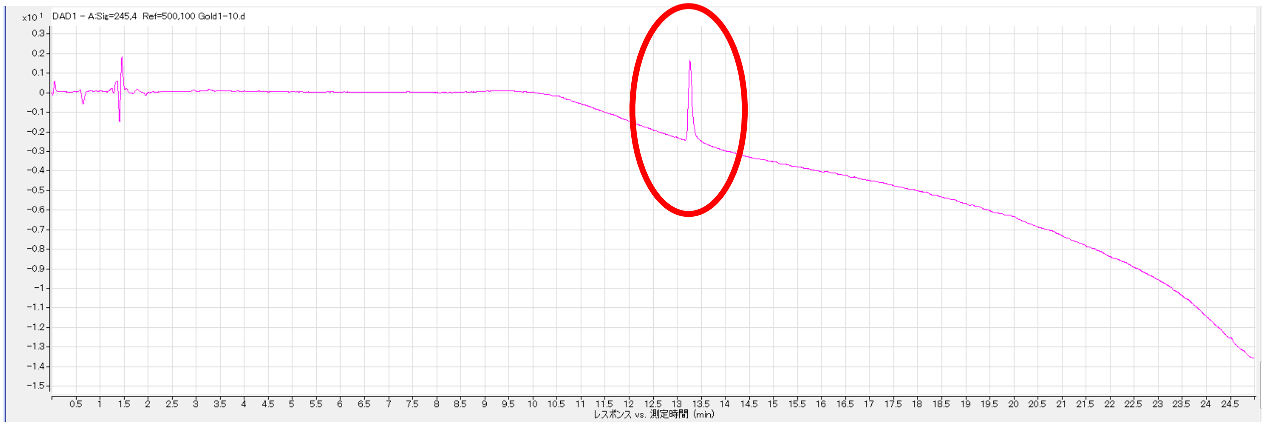

Figure S2

Figure S2. LC-UV detection of pigments

negative scan mode

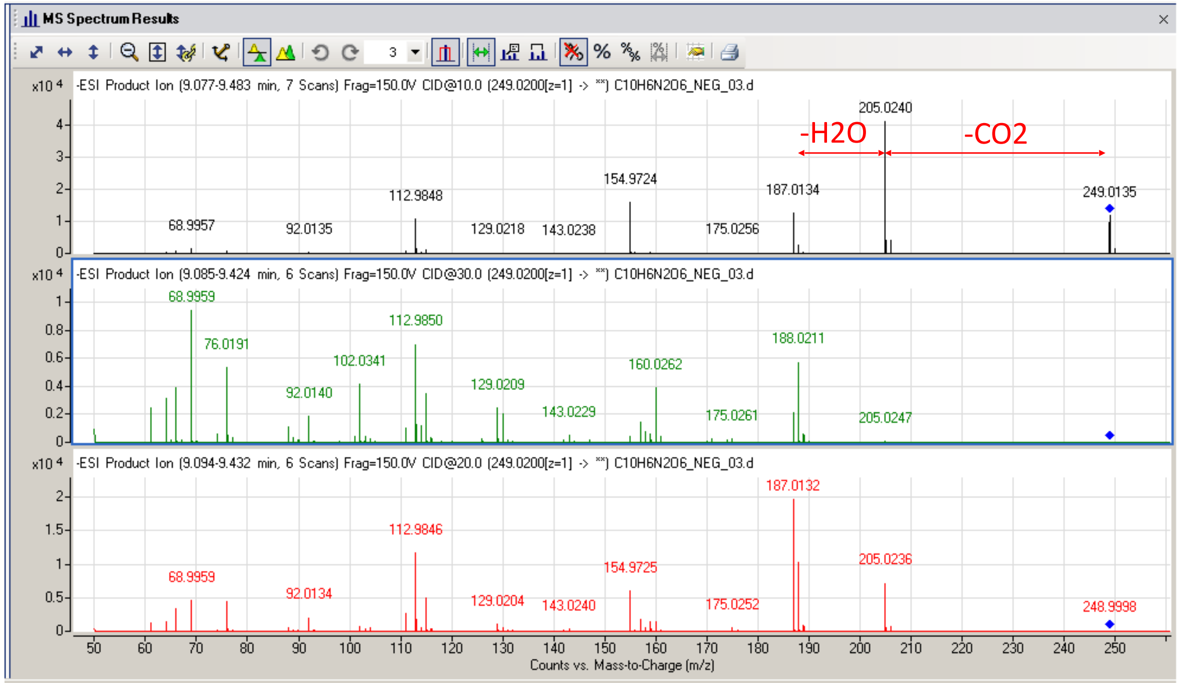

positive scan mode

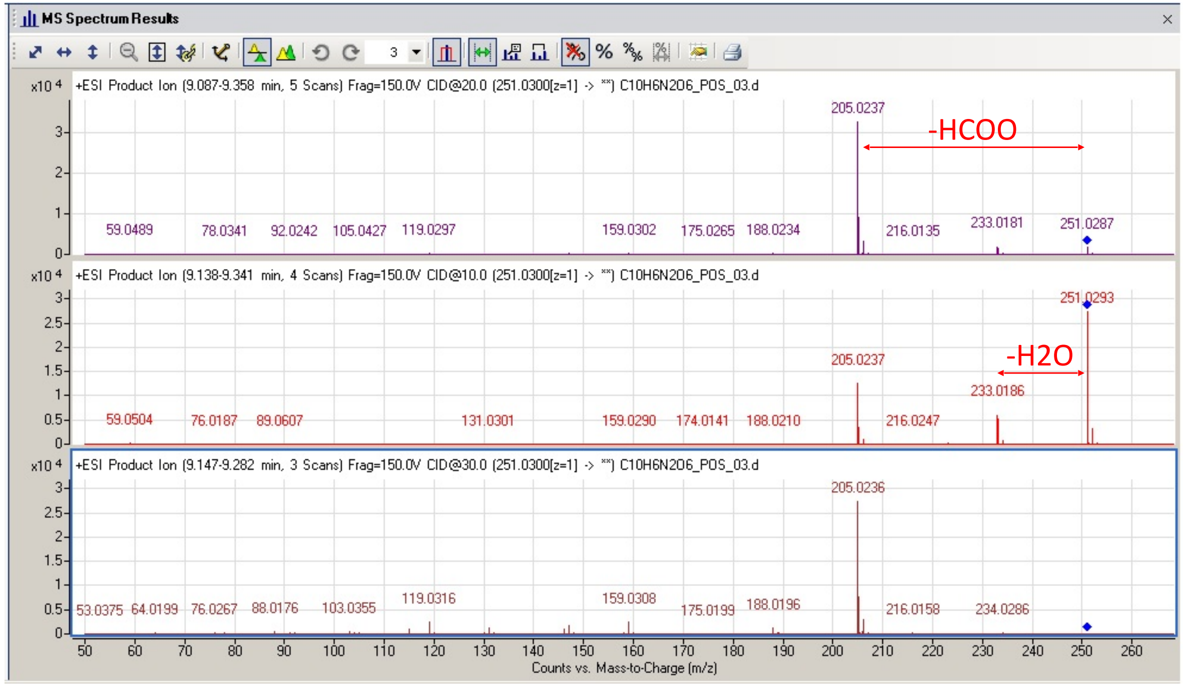

Figure S3

Figure S3. MS/MS spectra of $C_{10}H_6N_2O_6$ compound

A

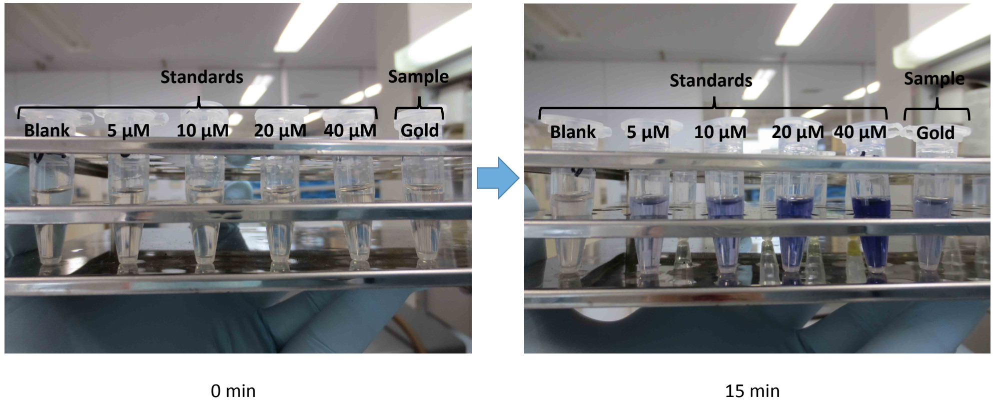

B

Sample	weight (mg)	extraction buffer (μL)	dilution	Concentration of sample (μM)	Concentration of XA (μM)
Gold	2.35	94	30	8.66	260

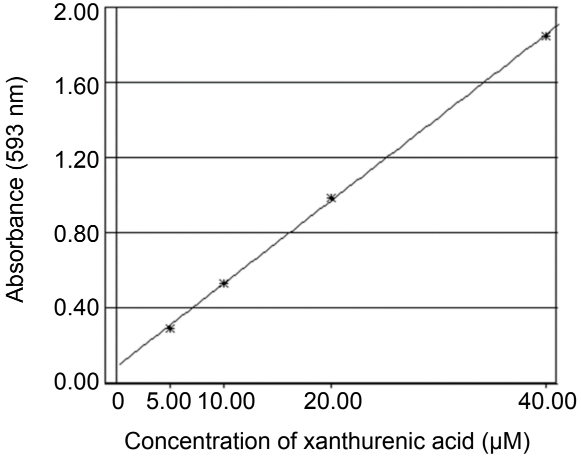

C

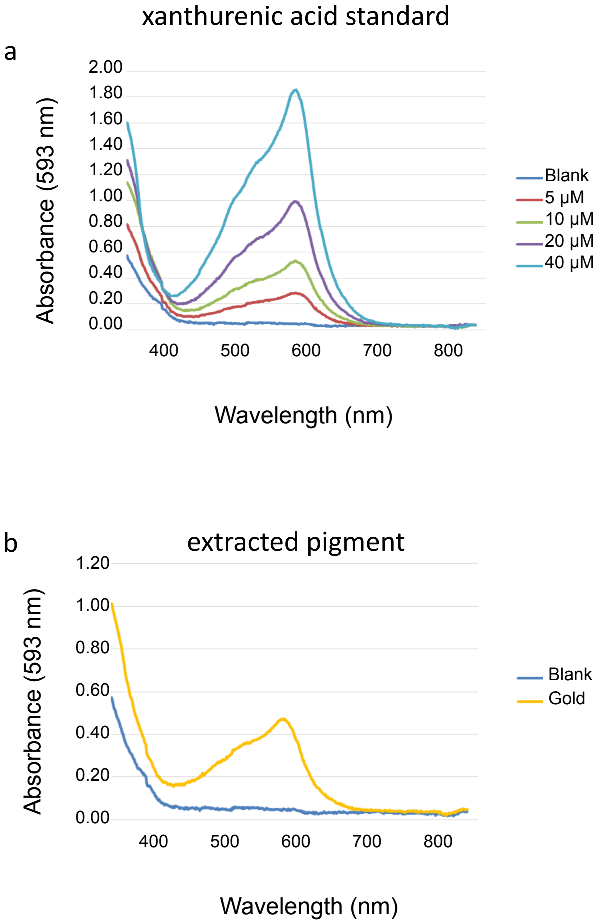

Figure S4. Quantitation of xanthurenic acid by reduction of Fe3+ - TPTZ complex

A. Reduction of Fe^{3+} - TPTZ complex by xanthurenic acid. From left, blank, 0, 5, 10, 20, 40 μM xanthurenic acid and sample. 0 min (left) and 15min (right) after the reaction. B. Quantitation of xanthurenic acid. C. Spectral scan for xanthurenic acid: Xanthurenic acid standard (a) and extracted pigment (b) .

Figure S4
